# Interoceptive Axes Dissociation in Anorexia Nervosa: A Single Case Study With Follow Up Post-recovery Assessment

**DOI:** 10.3389/fpsyg.2018.02488

**Published:** 2019-01-17

**Authors:** Daniele Di Lernia, Silvia Serino, Nicoletta Polli, Chiara Cacciatore, Luca Persani, Giuseppe Riva

**Affiliations:** ^1^Department of Psychology, Università Cattolica del Sacro Cuore, Milan, Italy; ^2^Applied Technology for Neuro-Psychology Lab, IRCCS Istituto Auxologico Italiano, Milan, Italy; ^3^UO di Endocrinologia e Malattie Metaboliche, IRCCS Istituto Auxologico, Milan, Italy; ^4^Dipartimento di Scienze Cliniche e di Comunità, Università degli Studi di Milano, Milan, Italy

**Keywords:** interoception, anorexia nervosa, interoceptive deficit, multisensory integration, body perception

## Abstract

Anorexia nervosa (AN) is a disorder characterized by alterations in body perception. Recent literature suggested that AN can also impair the processing of stimuli from inside the body (i.e., interoceptive) however, very few studies performed a complete interoceptive assessment exploring the evolution of the interoceptive dimensions before and after the subject’s recovery. To address this gap in knowledge, this study presented the case of Diana, a 25 years old woman affected by AN. At hospital admission, Diana performed a complete interoceptive assessment for accuracy (IAc), metacognitive awareness (IAw), sensibility (IAs), and interoceptive buffer saturation (IBs) – a new index that behaviourally evaluated the amount of interoceptive processing. Measures were repeated at the end of an outpatients rehabilitative hospital program, after Diana’s recovery. Results were confronted with a control (*N* = 4) of healthy female subjects. Analyses indicated severe deficits in accuracy, buffer saturation, and sensibility compared to control group. Conversely, metacognitive awareness was pathologically enhanced. After the rehabilitative hospital program, Diana’s clinical condition was largely improved and this reflected back on the interoceptive patterns that appeared restored, with no difference in interoceptive accuracy and metacognition compared to the control group. In conclusion, results indicated a very specific dissociation between interoceptive axes in AN with pervasive deficits in perception and processing that were accompanied by a pathologically enhanced confidence in the wrong perceptions. This case study reported an interesting and unique clinical pattern with a severe dissociation between interoceptive perceptions that nonetheless appeared restored after the subject’s recovery, highlighting the role of interoceptive assessment in the clinical evolution of AN.

## Introduction

The way we perceive our body defines our experience of life, our perception of self, and our well-being. Several disorders disrupt this perception and, among these, Anorexia Nervosa (AN) has been connected to profound alterations in our bodily experiences and representations (Cash and Deagle, 1997; Dakanalis et al., 2016). Further exploring alterations in body representation, Riva and Dakanalis (2018) recently proposed a model of AN that identified multisensory integration deficits as core elements of this clinical condition. Besides, the model is among the first to integrate signals from within the body (i.e., interoceptive signals) as pivotal inputs in the multisensory integration processes of AN, rediscovering the importance of interoception in relation to disturbances in body representation.

In the last decades, interoception received a broad new attention from neuroscience as a construct that encompasses all the physiological sensations from the entire organism (Craig, 2002, 2003, 2009). Several interoceptive alterations have been found in AN and neuroimaging evidence suggested that this pathology can compromise numerous key cortical structures within the interoceptive network. Specifically, AN subjects exhibited white matter abnormality and altered resting state functionality (Gaudio et al., 2015, 2017) of the anterior cingulate cortex (ACC), along with volume reduction of the right posterior insular cortex (Zucker et al., 2017) also in weight-restored AN patients (Kerr et al., 2016). Besides, AN alterations are not only limited to neurophysiological levels. Different studies (Pollatos et al., 2008; Fischer et al., 2016) indicated interoceptive behavioral deficits in AN subjects that specifically exhibited reduced interoceptive accuracy (i.e., the ability to correctly perceive inner body sensations) and reduced interoceptive sensitivity (i.e., self-reported beliefs about inner body perceptions assessed through questionnaires). Nevertheless, literature also reported contradictory evidence regarding AN and interoception. Ambrosecchia et al. (2017) found no differences in IAc between AN subjects and healthy controls. In a similar manner, Khalsa et al. (2015) reported comparable interoceptive results between AN subjects and healthy subjects in a modified interoceptive perception task. This evidence indicated a very complex relationship and this relationship is still missing several fundamental pieces of information due to the fact that interoceptive deficits in AN have not been systematically explored yet.

In particular, to the best of our knowledge, no previous evidence in literature explored the relationship between different interoceptive axes beyond accuracy and sensibility. Moreover, the same evidence did not explore how these deficits are related to each other and how they can vary across the clinical evolution of AN, providing an incomplete picture of a very complex interaction. It is, therefore, possible that AN is accompanied by a specific dissociation in the interoceptive dimensions, where deficits in perception reported in literature might also be accompanied by distortions in metacognitive awareness and stimuli processing, ultimately preventing the subject from resolving the bodily distortions that define the AN disorder.

To address this gap in knowledge, the study presented the case of a young AN woman with pervasive interoceptive alterations. The case-report investigated the interoceptive experience in a patient suffering from AN by using a complete interoceptive assessment repeated at different points in the clinical evolution of the pathology (i.e., admission and post-recovery). The aim of the current study was to report the role of the interoceptive dimensions in the clinical evolution of AN, highlighting the value of the different interoceptive facets and how they connected to each other across the temporal evolution of the disorder, providing support to the important role that the interoceptive assessment might have for the clinical evaluation of AN in the effort to integrate research and clinical practice (Fernandez-Alvarez et al., 2018).

## Case Presentation

Diana (a pseudonym) is a young woman of 25 years old that reached the Eating Disorders Centre, Division of Endocrine and Metabolic Diseases, San Luca Hospital in Milan, following a dramatic weight loss. Diana reached the Centre with a BMI of 16.06 kg/m^2^ reporting several disruptions in her eating patterns and several distressful alterations in her body image perception. As reported in the clinical history, Diana’s first eating related crisis was dated back 2 years before her current admission, with a subtle episode when she started a diet to lose some weight after health issues related to her thyroid. During that period, Diana was located abroad for work and – under moderate stress – she began a restrictive diet with a low caloric intake that brought her to lose 10 kg in 6 months. Diana’s weight remained constant in the following months but she developed an obsessive attention to the caloric intake along with intrusive thoughts regarding her weight and regarding specific types of foods. Diana also reported body-related image distortions such as overvaluation of her weight, mirror and body checking, and avoidance of body exposure. Moreover, during the crises, she reported frequent crying spells observing her body in front of a mirror.

In the last year, Diana reported a stressful situation at the University that heightened her psychological symptoms. Following these new difficulties, Diana re-enacted the restrictive conducts, reducing the caloric intake with a consequent weight loss of 4 kg in a month. In the period before the admission, the restrictive conduits were accompanied by self-induced vomit and daily binge episodes.

### Case Formulation

Diana matched all the DSM-5 criteria for a diagnosis of AN, binge-purge subtype. Compatible with the diagnosis, Diana presented distortions in her body perception and obsessive thoughts regarding her weight and her body image; for these reasons, she was considered as an optimal candidate for the interoceptive assessment.

At the begin of the rehabilitative protocol, Diana’s blood panels showed no signs of metabolic distress, with values in normal ranges. At her admission, Diana’s thyroid levels were within normal range and they remained within the normal range during the curse of the treatment. Endocrinologist suggested a chronic autoimmune normal-functioning condition. The psychiatric assessment indicated mood alterations toward a depressive condition accompanied by severe sleep difficulties and insomnia.

### Course of Treatment

The rehabilitative program was composed of a multidisciplinary approach that included several experts in different fields: endocrinology, psychiatry, psychology, and nutrition. The specialists collaborated in an outpatients service tailored to the specific users’ needs. The rehabilitative program could extend from two to four cycles of treatment. Diana followed a two-cycle rehabilitative protocol with a frequency of 3 sessions a week for a total of 37 sessions. The protocol was composed of psychological intervention with group psychotherapy and individual sessions focused upon a psyco-corporal therapy approach (body-oriented psychotherapy). Psychological intervention was accompanied by psychiatric and pharmacological support (citalopram and mirtazapine), to moderate Diana’s mood alterations. The rehabilitative protocol was integrated with alimentary education sessions provided by the nutritionist. Additionally, the protocol was also accompanied by a nutritional program with fixed meals (both in quantity and composition) that Diana consumed under supervision. Scheduled assessment sessions ensured an adequate monitoring of the progress.

From the beginning, Diana showed a deep insight regarding her condition. Nonetheless, from her first session, Diana showed severe difficulties in following the assigned rehabilitative diet due to obsessive thoughts connected to her body weight and to certain types of food. In the following weeks, Diana improved her adherence to the recovery protocol with a better ability to follow the changes in the diet both on quantitative both on the qualitative level (e.g., types of foods consumed). Diana reached a BMI of 19.00 kg/m^2^ at the end of her second cycle of rehabilitative treatment. Considering the noticeable improvements regarding her eating behaviors and her general clinical condition, Diana was dismissed from the Centre and continued her program following only monthly assessments accompanied by individual psychotherapy.

## Measures, Investigation, and Methods

To evaluate possible interoceptive distortions, Diana performed a completed interoceptive assessment at her hospital admission and after the rehabilitative program. Following literature suggestion for single case analysis (Crawford and Howell, 1998; Crawford et al., 2004, 2006, 2009), Diana’s performances were compared to healthy subjects control group. Written informed consent was obtained from the patient both for the purposes of research participation as well as for the publication of this research report, including their identifiable information.

### Healthy Subjects Control Group

Four healthy female subjects matched for age [mean = 24.75; *SD* = 4.11] were recruited with consecutive sampling as a part of a larger enlisting procedure. Exclusion criteria were the presence of current psychological or physical diagnoses, the presence of subclinical conditions relate to AN, and presence of subclinical depressive conditions. The risk for eating-related disorders was assessed in the healthy sample with EDI-3 Drive for Thinness subscale, with a cut-off of <7 (Garner et al., 1983; Clausen et al., 2011; Eshkevari et al., 2012). Depressive mood disorders were assessed through the Beck Depression Inventory (BDI-II) with a cut-off of <13 (Beck et al., 1961; Steer et al., 1999; Storch et al., 2004). Control subjects received instruction to avoid medications in the 12 h before the meeting, and nicotine and caffeine in the 2 h before the experiment. All subjects gave written informed consent in accordance with the Declaration of Helsinki (2008). The protocol was approved by the Ethics Committee of Catholic University of Sacred Heart of Milan.

### Interoceptive Measures

Interoceptive Awareness (IA) is a construct which describes behavioral, metacognitive, and cognitive processes related to inner body perceptions. The subcomponents of IA are interoceptive accuracy, interoceptive metacognitive awareness, and interoceptive sensibility (Garfinkel et al., 2015).

#### Interoceptive Accuracy

Interoceptive accuracy (IAc) is a behavioral index that reports how much a subject is able to correctly perceive inner body sensations, and it is most commonly measured through the heartbeat perception task (Schandry, 1981). Alterations in this index have been connected to a variety of conditions both on clinical and non-pathological levels (Di Lernia et al., 2016b, 2018c) and also to AN (Pollatos et al., 2008; Fischer et al., 2016). The task asks the subjects to silently count their heartbeats focusing only on inner body sensations. Subjects have therefore to count their heartbeats in specific time span (25, 35, and 45 s) and reported heartbeats are compared to real heartbeats measured through an ECG. Index score can vary between 0 and 1, where lower values indicate poorer performances.

#### Interoceptive Metacognitive Awareness

Interoceptive metacognitive awareness (IAw) expresses a metacognitive confidence in the subject’s heartbeat task perception (Garfinkel et al., 2015). In the task, subjects are invited to give a response of confidence regarding their performance at the accuracy task. Responses are given on a Visual Analog Scale that ranges from 0 (Not Confident at All) to 100 (Fully Confident), without any feedback on the actual performance.

#### Interoceptive Sensibility

Interoceptive sensibility (IAs) expresses the subject’s cognitive beliefs regarding body perceptions. IAs is measured through self-reported questionnaires, among which the Multidimensional Assessment of Interoceptive Awareness (MAIA) questionnaire (Mehling et al., 2012). The MAIA is a multi-dimensional 32 items questionnaire with 8 subscales. The Noticing (NO) subscale expresses subject’s awareness of uncomfortable, comfortable, and neutral body sensations. The Not-Distracting (ND) subscale expresses subject’s tendency not to ignore or distract oneself from sensations of pain or discomfort. The Not-Worrying (NW) subscale expresses subject’s tendency not to worry or experience emotional distress with sensations of pain or discomfort. The Attention Regulation (AR) subscale expresses subject’s ability to sustain and control attention to body sensations. The Emotional Awareness (EA) subscale expresses subject’s awareness of the connection between body sensations and emotional states. The Self-Regulation (SR) subscale expresses subject’s ability to regulate distress by attention to body sensations. The Body Listening (BL) subscale expresses subject’s ability to active listening to the body for insight. The Trusting (TR) subscale expresses subject’s experience of one’s body as safe and trustworthy. Responses are given on a 6 points likert scale, from 0 to 5. Each subscale score ranges from 0 to 5.

#### Interoceptive Buffer Saturation Index

The interoceptive buffer saturation index (IBs) is a recently developed index (Di Lernia et al., 2016a, 2018c) that measures levels of interoceptive processing^1^ through a verbal time estimation of interoceptive tactile stimuli delivered with a specific stimulator (Di Lernia et al., 2018a,b).

Interoceptive tactile stimuli are a secondary kind of tactile inputs that directly report to the insular cortex and are connected to differ aspect of body perception also in AN (Roudaut et al., 2012; Gordon et al., 2013; Ackerley et al., 2014; Crucianelli et al., 2016).

From this perspective, the IBs index utilizes verbal time estimation of interoceptive tactile stimuli, to reversely evaluate the amount of interoceptive processing through distortions in the time perception of the stimuli according to Craig’s emotional asymmetry (Craig, 2009) temporal hypothesis – for further details see Di Lernia et al. (2018c).

From a methodological point of view, the task delivers several interoceptive tactile stimuli to the subject’s left volar forearm subsequently asking for verbal time estimations of the duration of the stimuli. The interoceptive tactile stimulation is composed by low-velocity low-force (i.e., 3 cm/s; 2.5 mN) tactile brushing stimuli of six fixed durations (8, 10, 12, 14, 16, and 18 s) randomly repeated for 3 blocks, for a total of 18 stimuli. Partial accuracy indexes for each time interval is calculated with the following formula: 1/3Σ [(time estimation - real time)/real time]. Total index is calculated as mean of partial indexes and lower scores indicate a lower amount of interoceptive processing.

### Statistical Analyses

Statistical analyses were performed according to Crawford and Garthwaite (2002) and Crawford and Howell (1998) using a modified *t*-test for comparison of single case results with small normative samples. Crawford and Garthwaite (2002) methodology offered a robust statistical choice for non-normal distributions, with low type I error values. Following literature suggestions (Crawford and Garthwaite, 2002; Crawford et al., 2004, 2006, 2009; Couto et al., 2013) effect sizes are reported as point estimates (z-cc for the modified *t*-test). Analyses were conducted with Singlims_ES (Crawford and Garthwaite, 2002).

## Results

### Sample Psychological Characteristics and Measures

The healthy female control group showed normal levels of interoceptive accuracy [mean = 0.55; *SD* = 0.11] comparable to previous studies (Garfinkel et al., 2015; Di Lernia et al., 2018c). Metacognitive awareness [mean = 55.81; *SD* = 13.82] was aligned with accuracy, as also found in the previous literature (Garfinkel et al., 2015). Healthy subjects’ IAs scores were in range with literature (Cali et al., 2015), see Table 1. Furthermore, control group showed no risk of Drive for Thinness tendencies [mean = 2.75; *SD* = 3.20] and normal mood variation as reported by the BDI-II [mean = 6.5; *SD* = 5.00]. Moreover, the sample reported no risk of Bulimia tendencies [mean = 2.25; *SD* = 1.89] and low risk of body dissatisfaction [mean = 11.25; *SD* = 5.85]. Results are summarized in Table 1.

**Table 1:** Sample characteristics, psychological, and interoceptive measures.

	Diana		Control group	
		
	*Baseline*	*Post-Recovery*	*Mean*	*SD*
Age	25	25	24.75	4.11
BMI	16.06	19.00	19.8	1.62
EDI_DT	21^b^	0^b^	2.75	3.20
EDI_B	2^b^	0^b^	2.25	1.89
EDI_BD	16^b^	6^b^	11.25	5.85
STAI_T	65	62	37.00	6.32
STAI_S	32	30	29.75	0.98
IAc	0.08^∗^	0.55	0.55	0.11
IAw	93.67^∗a^	70.66	55.81	13.82
IBs	-0.47^∗^	-0.36	0.60	0.30

**MAIA subscales**				

Noticing	3	4	3.18	1.26
Not-Distracting	1.33	1^∗a^	2.08	0.32
Not-Worrying	3	2	3.25	0.95
Attention Regulation	1.85	2.57	2.46	0.76
Emotional Awareness	3	2.60	3.80	0.58
Self-Regulation	0.75^∗a^	0.75^∗a^	2.43	0.55
Body-Listening	1.33	0.66	2.91	0.95
Trusting	1.66^∗^	1.00^∗^	3.75	0.57


*^∗^Difference is significant at level 0.05 (two tails). ^∗*a*^Difference is significant at level 0.05 (one tail). BMI, body mass index; EDI_DT, Drive for Thinness risk subscale; EDI_B, Bulimia risk subscale; EDI_BD Body Dissatisfaction risk subscale. ^*b*^the patient’s scores were collected with the EDI-2. STAI_T, STAI trait anxiety; STAI_S, STAI state anxiety; IAc, interoceptive accuracy; IAw, interoceptive metacognitive awareness; IBs, interoceptive buffer saturation index.*

### Interoceptive Measures

At the baseline, Diana showed a significantly lower interoceptive accuracy compared to healthy controls subjects (IAc) [*t* = -3.824, *p* = 0.031, Z-CC = -4.276], along with a significantly lower buffer saturation (IBs) [*t* = -3.162, *p* = 0.05, Z-CC = -3.536]. Interestingly, metacognitive awareness (IAw) was significantly enhanced compared to healthy subjects [*t* = 2.450, *p* = 0.045, Z-CC = 2.739]. The reported differences were not found at post-recovery assessment. At the baseline, Diana showed significant differences in interoceptive sensibility (IAs) as assessed by the MAIA questionnaire. Specifically, compared to the control group^2^ she was less able to regulate distress through attention to body sensations [Self-Regulation subscale, *t* = -2.723, *p* = 0.036, Z-CC = -3.044] and she was experiencing her body less safe and trustworthy than healthy subjects [Trusting subscale, *t* = -3.275, *p* = 0.046, Z-CC = -3.661]. At post-recovery assessment, Self-Regulation difference remained unaltered, while Trusting scores diminished even more [*t* = -4.321, *p* = 0.022, Z-CC = -4.831]. Moreover, Diana showed a significant difference [*t* = -3.037, *p* = 0.027, Z-CC = -3.396] in the Not-Distracting subscale compared to the control group, indicating that she was less able to distract herself from distressful body sensations. Results are summarized in Table 1.

## Case Discussion and Conclusion

The study presented a single case analysis of Diana, AN subject with pervasive interoceptive distortions. Before the rehabilitative program, Diana’s ability to correctly perceive her inner body signals (interoceptive accuracy) was severely distorted and diminished with extremely low scores that approximated to zero, indicating an extremely poor ability to correctly perceive inner body sensations. In the same way, the interoceptive buffer saturation index – a recently developed interoceptive index that is able to infer the amount of interoceptive processing – indicated that Diana was processing a significantly lower amount of interoceptive signals compared to a control group of healthy subjects. These results confirmed and further extended previous literature evidence that identified interoceptive deficits in AN (Gaudio et al., 2015, 2017; Wierenga et al., 2015; Fischer et al., 2016; Kerr et al., 2016) nonetheless Diana’s case presented an interesting interoceptive pattern that, to the best of our knowledge, has never been previously described in literature.

Diana’s ability to correctly perceive her inner body sensations was severely compromised nevertheless, she was – at the same time – deeply convinced that her distorted perceptions were correct. Diana’s metacognitive awareness indicated an almost complete confidence (IAw score 93 over 100) in the compromised perceptions, thus suggesting a profound detachment between Diana’s ability to perceive her body and the awareness of her deficits.

In the healthy subjects control group, there was strong alignment between the ability to correctly perceived inner body sensations (accuracy) and the awareness of these perceptions (metacognitive awareness), as also confirmed by literature that indicated a positive correlation between these two dimensions (Garfinkel et al., 2015). Nonetheless, Diana’s case presented a pervasive cognitive split between real bodily perceptions and the awareness regarding these sensations, where a poor perception of bodily signals was accompanied by an extremely high confidence in these diminished perceptions.

Interoceptive sensibility – a construct related to Diana’s cognitive beliefs regarding her body – indicated a significative difference in the Trusting subscale at the moment of her hospital admission, suggesting that Diana perceived her body less safe and trustworthy than healthy female subjects. Moreover, Diana’s scores approached significance for a diminished ability to regulate distress by posing attention to body sensations (Self-Regulation subscale) along with a diminished ability to active listening to the body for insight (Body Listening subscale), a result that deeply connects with Diana’s deficits in perception and in processing of interoceptive stimuli.

A recent work by Brown et al. (2017) provided normative interoceptive sensibility values for subjects with eating disorders. Compared to a complementary subgroup with AN binge-purge subtype, Diana’s scores showed a similar pattern with deficits along the same directions identified by Brown. Diana’s deficits in the self-regulation, the body listening, and the trusting subscales were aligned with Brown’s evidence, which identified these scales as the most sensitive to alterations connected to eating disorders. Notably, Diana’s scores for all the MAIA subscales were generally lower than the normative values presented in Brown et al. (2017) both at admission and at post-recovery assessment, suggesting a pervasive interoceptive alteration that highlights the unique value of the presented case study.

Before the rehabilitative program, Diana’s interoceptive perceptions exhibited a specific dissociation connected to a disrupted alignment between the actual bodily sensations and the metacognitive awareness of these sensations. This particular dissociation suggested a distinctive multisensory impairment (Riva, 2018) that could be connected to Diana’s difficulty to coherently integrate input arising from within the body with the metacognitive perceptions regarding the body itself. This evidence supports a recent framework proposed by Riva and Dakanalis (2018) further suggesting a distinctive pattern in AN characterized by multisensory integration deficits that are able to impair the subject’s ability to “correctly link internal (interoceptive) bodily signals to their potential pleasant (or aversive) consequences” (Riva and Dakanalis, 2018).

After the rehabilitative program, Diana presented an almost completely repaired interoceptive pattern. Interoceptive accuracy, the ability to correctly perceive sensations arising from the body, appeared restored and Diana’s score did not report any significant difference compared to the control group reaching a value that was also in range of other non-clinical samples reported in literature (Pollatos et al., 2008; Di Lernia et al., 2018c). In a similar manner, there was no difference in the amount of interoceptive inputs processed by Diana compared to healthy subjects, as reported by the interoceptive buffer saturation index. Furthermore, Diana’s metacognitive awareness indicated a healthy alignment process, as reported by the change in the metacognitive perception that shifted toward the real perception, reducing the distorted and pathologically enhanced confidence emerged in the initial assessment. This interesting result indicated that the extreme gap between Diana’s perception and awareness of bodily sensations was partially and functionally restored at the follow-up assessment when Diana was not only able to correctly perceive her inner bodily sensations but she was also able to better evaluate the correctness of those perceptions.

Regarding body cognitive beliefs collected by the MAIA subscales, Diana showed an increased tendency to worry about sensations of discomfort after the rehabilitative protocol. Moreover, Diana’s exhibited low interoceptive trusting scores compared both to the beginning of the treatment both to the control group.

These very interesting patterns suggest that a restored interoceptive perception, along with a functional interoceptive processing and an improved metacognitive awareness, might have further compromised Diana’s body perception because those sensations that were interpreted as correct during the acute phase of the disorder were now revealed as false and inaccurate. Although apparently paradoxical, results connected to Diana’s trust in her body can be considered as a break in the pathological perception of an altered body representation, also considering the outcome of the rehabilitative program, when a positive and restored BMI was accompanied by profound and positive changes in Diana’s eating behaviors and emotional regulation. These positive changes were reflected by the EDI Drive for Thinness and Body Dissatisfaction scores that reached non-pathological levels at the end of the second cycle of treatment, suggesting that Diana’s body image perception was also improved post-treatment.

Nonetheless, several other possible interpretations of the results must be considered. As a matter of fact, it is also possible that Diana’s lower scores in MAIA Trust subscale at post-recovery assessment could be simply due to the positive variation in her weight, which was inevitably connected to a pervasive change in the way Diana experienced her body. As reported by the BMI positive variation, Diana’s body objectively changed over the course of treatment and it is therefore probable that this very change could have disrupted Diana’s trust in her own body also considering that weight gain in AN is also frequently associated with an increase in body image concerns.

Even so, before the treatment, Diana evaluated her distorted and diminished interoceptive sensations as correct with an almost complete confidence because her perceptive deficits did not reach cognitive awareness. Following a successful recovery – as also indicated by the positive variation in BMI, EDI scores, and clinical evaluation by the multidisciplinary team that followed the case – the restored interoceptive abilities opened a new perspective in Diana’s body perception and it is possible that these new healthy and functional perceptions were accompanied by a disruptive impact on the subject’s pathological beliefs. Therefore, we could hypothesize that – under Diana’s point of view – the same body that was considered as a reliable source of information before the rehabilitative program was later experienced less trustworthy, due to the awareness that those sensations that misguided Diana’s life for 2 years were later revealed false and inaccurate. Ultimately, it is possible that this process created a new body perception that the young woman had to explore, reconcile, and re-experience as her own.

The results presented so far suggest several implications for the clinical practice, although more data need to be collected with larger samples to generalize the conclusions presented. Notwithstanding, Diana’s case report highlighted the value of a complete interoceptive assessment in AN as a diagnostic instrument to evaluate the severity of the dissociation between the actual bodily perceptions and the pathological metacognitive beliefs that the patient might cultivate. The nature of the interoceptive dissociation, as presented in Diana’s clinical evolution can, therefore, provide useful information for the clinician to better address the recurrent perceptive distortions that are pivotal elements of the disorder. Moreover, the complex relationship between interoceptive perceptions and AN suggests the possibility to address this specific disorder through interoceptive related treatments. Interoceptive treatments are bodily focused integrated treatments based on the Embodied Cognition framework and the concept of multisensory integration (Riva et al., 2017; Riva and Dakanalis, 2018). They have been used to reduce symptoms severity in somatoform disorders (Schaefer et al., 2014), and they have recently been applied to different conditions with promising results (Watson et al., 2018). Technology can be helpful in this process (Azevedo et al., 2017; Riva and Gaudio, 2018) too. For example, “sonoception” (www.sonoception.com), a novel noninvasive technological paradigm based on wearable acoustic and vibrotactile transducers able to stimulate mechanoreceptors in different parts of the body, was recently used to assess interoceptive time perception (Di Lernia et al., 2018c) and to enhance heart rate variability (the short-term vagally mediated component—rMSSD) through the modulation of the subjects’ parasympathetic system (Di Lernia et al., 2018a). From this perspective, considering the relationship between the interoceptive perceptions and the clinical evolution of the pathology, as highlighted in the case report, the clinical practice might benefit from the possibility to use interoceptive assessment and interoceptive treatments to address the complexity of AN.

## Author Contributions

All authors listed have made a substantial, direct and intellectual contribution to the work, and approved it for publication.

## Conflict of Interest Statement

The authors declare that the research was conducted in the absence of any commercial or financial relationships that could be construed as a potential conflict of interest.

## Footnotes

1 In addition, previous evidence indicated that the IBs index is able to discriminate low interoceptive processing connected to EDI-3 Drive for Thinness tendencies and eating-related disorders risks (Di Lernia et al., 2018c).

2 Although very few studies analyzed IAs with longitudinal MAIA measures in healthy subjects, recent evidence indicated that MAIA subscales scores did not change significantly for healthy subjects, across a time window of more than 3 months (Bornemann et al., 2015). In a similar manner, interoceptive accuracy has been proven quite stable across time in healthy participants, specifically the Schandry task demonstrated a good test–retest reliability, with scores from 0.58 (Mussgay et al., 1999) up to 0.80 (Werner et al., 2013). This specific time consistency provide useful comparison for Diana’s pre and post-recovery scores.
